# Quantum-like model of unconscious–conscious dynamics

**DOI:** 10.3389/fpsyg.2015.00997

**Published:** 2015-08-03

**Authors:** Andrei Khrennikov

**Affiliations:** Department of Mathematics, Mathematical Institute, Linnaeus UniversityVäxjö, Sweden

**Keywords:** sensation, perception, quantum-like model, quantum apparatuses and instruments, bistable perception, unconscious inference

## Abstract

We present a quantum-like model of sensation–perception dynamics (originated in Helmholtz theory of unconscious inference) based on the theory of quantum apparatuses and instruments. We illustrate our approach with the model of bistable perception of a particular ambiguous figure, the Schröder stair. This is a concrete model for unconscious and conscious processing of information and their interaction. The starting point of our quantum-like journey was the observation that perception dynamics is essentially contextual which implies impossibility of (straightforward) embedding of experimental statistical data in the classical (Kolmogorov, 1933) framework of probability theory. This motivates application of nonclassical probabilistic schemes. And the quantum formalism provides a variety of the well-approved and mathematically elegant probabilistic schemes to handle results of measurements. The theory of quantum apparatuses and instruments is the most general quantum scheme describing measurements and it is natural to explore it to model the sensation–perception dynamics. In particular, this theory provides the scheme of indirect quantum measurements which we apply to model unconscious inference leading to transition from sensations to perceptions.

## 1. Introduction

In recent years the mathematical formalism of quantum mechanics was applied to a variety of problems outside of quantum physics: from molecular biology and genetics to cognition and decision making (see the monographs, Khrennikov, 2010b; Busemeyer and Bruza, 2012; Haven and Khrennikov, 2012) and the extended lists of references in them as well as in the papers (Aerts et al., 2014; Khrennikov et al., 2014).

The problem of mathematical modeling of bistable perception and, more generally, unconscious inference^1^ is that it can be rather complex and that its nature is not understood well-enough to allow one to choose the optimal model. In spite of tremendous efforts during the last 200 years, this problem cannot be considered fully solved (cf. Newman et al., 1996; Laming, 1997). In this note we apply the theory of *quantum apparatuses and instruments* (Davies and Lewis, 1970; Busch et al., 1995; Ozawa, 1997) to quantum-like modeling of sensation–perception dynamics as the concrete example of *unconscious and conscious processing of information and their interaction.* Our model can be applied to general unconscious–conscious information processing. It generalized the quantum-like model developed in Khrennikov (2004). We also point out that this paper is the first attempt to apply the theory of quantum apparatuses and instruments outside of physics, to cognition and psychology.

Special quantum structures were elaborated in order to mathematically represent most general measurement schemes and are applicable both in classical and quantum physics and, practically, in any domain of science. They generalize the pioneer quantum measurement representation by operators of the projection type, also known as von Neumann–Lüders measurements. In quantum physics, this new general framework is of vital importance since the projection type measurements do not completely cover real experimental situations (Davies and Lewis, 1970; Busch et al., 1995; Ozawa, 1997; Nielsen and Chuang, 2000). It seems that the same holds true in mathematical modeling in cognition and psychology (see Asano et al., 2010a,b; Khrennikov, 2010b; Asano et al., 2011, 2012; Khrennikov and Basieva, 2014; Khrennikov et al., 2014), although here the situation is not yet absolutely clear and, obviously, the underlying reason for using quantum instruments is different.

To motivate the use of the theory of quantum apparatuses and instruments, we shall compare it first to classical probabilistic methods and then to simpler quantum-like models of processing data from cognitive science and psychology based on the von Neumann–Lüders measurements. A detailed discussion on violation of laws of classical probability theory by statistical data collected in cognitive science and psychology can be found in Khrennikov, 2010b and SS. We can, for example, point to the order effect (Khrennikov, 2010b; Wang and Busemeyer, 2013) and the disjunction effect (Khrennikov, 2010b; Busemeyer and Bruza, 2012). In the probabilistic terms these are just various exhibitions of violation of the formula of total probability. In general, during recent years quantum probability and decision making were successfully applied to describe a variety of problems, paradoxes, and probability judgment fallacies, such as Allais paradox (humans violate Von Neumann–Morgenstern expected utility axioms), Ellsberg paradox (humans violate Aumann–Savage subjective utility axioms) (see e.g., Haven et al., 2009; Asano et al., 2010a,b, 2011, 2012; Busemeyer et al., 2011; Pothos and Busemeyer, 2013; Wang and Busemeyer, 2013; Aerts et al., 2014; Khrennikov and Basieva, 2014). Psychologists and economists explore the new way inspired by one simple fact from physics: *quantum probability can work in situations where classical probability does not.* Why? Answers may differ (see Khrennikov, 2010b). We point to contextuality of data as one of the main sources of its non-classicality (Khrennikov, 2010b; Dzhafarov and Kujala, 2012a,b, 2013).

As was pointed out, at the beginning of quantum theory physicists attempted to represent quantum measurements they were dealing with by projectors. The same attitude could be observed in applications of the quantum formalism outside of physics. Granted, some statistical psychological effects can be nicely described with the help of the von Neumann–Lüders measurements (see e.g., Haven et al., 2009; Busemeyer et al., 2011; Busemeyer and Bruza, 2012; Pothos and Busemeyer, 2013; Wang and Busemeyer, 2013; Aerts et al., 2014). However, more detailed analysis showed (Asano et al., 2010a,b; Khrennikov, 2010b; Asano et al., 2011, 2012; Khrennikov and Basieva, 2014; Khrennikov et al., 2014) that, in general, data from cognitive psychology cannot be embedded into the projection-measurement scheme. Therefore, it is natural to follow the development of quantum physics and proceed within a general theory of measurements.

In this paper we do this by illustrating the general theory of quantum instruments with one concrete example: *bistable perception* of the concrete ambiguous figure, *the Schröder stair.* Why do we use a quantum-like model? Here the argument is more complicated than in the case of the order and disjunction effects and other probability fallacies mentioned above. The deviation from classical probability theory is expressed not as a violation of the *formula of total probability*, but as a violation of one of the *Bell-type inequalities*, namely, the Garg–Leggett inequality (Asano et al., 2014). We point out that the Bell-type inequalities play an important role in modern quantum physics. If such an inequality is violated, then the data cannot fit a classical probability space. As was shown in our previous study (Asano et al., 2014), the data collected in a series of experiments performed at Tokyo University of Science (see Asano et al., 2014) for details, violate the Garg–Leggett inequality (statistically significantly)^2^.

The first step toward creation of a quantum-like model of bistable perception was done by Atmanspacher and Filk (2012, 2013). We studied this problem in Asano et al. (2014), where we demonstrated a violation of the Garg–Leggett inequality for experimental probabilistic data collected for rotating image of Schröder stair (the experiment was performed at Tokyo University of Science), in Accardi et al. (in press) we presented a quantum-like adaptive dynamical model for bistable perception. The latter is based on a more general formalism than the theory of quantum instruments—on the theory of adaptive quantum systems. In the present paper, the traditional approach to quantum measurement theory is used for modeling sensation–perception transition and unconscious inference.

Finally, we point out that violation of laws of classical probability theory is a statistical exhibition of violation of laws of classical Boolean logic. Thus, in logical terms the quantum-like modeling of cognition is modeling of a nonclassical reasoning, decision making, and problem solving. In particular, in our model unconscious inference, generation of a perception from a sensation, is not based on the rules of classical logics. We also remark that the so called quantum logic corresponding to the quantum formalism is just one special type of nonclassical logic. In principle, there are no reasons to assume that human (mental) cognition, even if it has a non-Boolean structure, can be modeled completely with the aid of quantum logic and quantum probability. Still more general models might be explored, see (Khrennikov and Basieva, 2014) for a discussion.

## 2. Advantageousness of quantum instrumental modeling in cognitive psychology

We emphasize that, as well as quantum physics (Plotnitsky, 2006, 2009), cognitive and social sciences also can be treated as theories of measurements. A great deal of effort has been put into the development of measurement formalisms, cf. with, e.g., the time-honored *Stimulus–Organism–Response (S–O–R) scheme* for explaining cognitive behavior (Woodworth, 1921). Just like the situation in quantum physics, cognitive and social scientists cannot approach the mental world directly; they work with results of observations. Both quantum physics and cognitive and social sciences are fundamentally based on operational formalisms for observations.

The basic notions of the operational formalism for the quantum measurement theory are *quantum apparatus and instrument* (Davies and Lewis, 1970; Busch et al., 1995; Ozawa, 1997). Quantum apparatuses are mathematical structures representing at a high level of abstraction physical apparatuses used for measurements. They encode the probabilities of the results of observations as well as the back-actions of the measurements on the states of physical systems. Such back-actions are mathematically represented with the aid of another important mathematical structure, a quantum instrument. Our aim is to explore the theory of quantum apparatuses and instruments and especially its part devoted to *indirect measurements* in cognitive and social sciences.

The scheme of *indirect measurements* is very useful for applications, both in quantum physics and humanities. In this scheme, besides the “principle system” *S*, a probe system *S*′ is considered. A measurement on *S* is composed of the unitary interaction with *S*′ and a subsequent measurement on the latter.

In our cognitive modeling *S represents unconscious information processing and S*′ *conscious.* In the concrete example of Helmholtz unconscious inference, *S* represents processing of sensation (its unconscious nature was emphasized already by Helmholtz) and *S*′ represents processing of perception - conscious representation of sensation.

This approach provides a possibility to extend the class of quantum measurements which originally were only von Neumann–Lüders measurements of the projection type. Such an extension serves not only the natural seeking of generality. Generalized quantum measurements have some new features. Here we shall concentrate only on those of them relevant to our project on quantum-like cognition.

For us, one of the main problems of exploring solely projective (direct) measurements is their fundamentally invasive nature: *as the feedback of a measurement, the quantum state is “aggressively modified”—it is projected onto the subspace corresponding to the result of this measurement*. In any event, this feature is not so natural for the dynamics of sensation and perception states. Of course, each “perception–creation” modifies the states of sensation and perception, *but these modifications are not of the collapse type*, as they should be in the case of projections.

Important for our applications is that *a variety of different quantum instruments (describing back-reaction transformations resulting from measurements) can correspond to one and the same observable* on the principle system *S*. That is, measurements having the same statistical results may lead to very different state transformations (due to very different types of interaction between the principle and probe systems). In quantum mechanics (as Ozawa emphasized Ozawa, 1997), the same observable can be measured by different apparatuses having different state-transforming quantum instruments. This is a very important characteristic of the theory of generalized quantum measurements. It is also very useful for cognitive modeling, since it reflects the individuality of measurement apparatuses/instruments which are used by cognitive systems (e.g., human beings) to generate the same perception.

We point out that the scheme of indirect measurements accounts for state dynamics in the process of measurement, which is not just a “yes”/“no” collapse as in the original von Neumann–Lüders approach. The possibility to mathematically describe the mental state dynamics in the process of perception–creation by means of the quantum formalism is very attractive. A study in this direction was already presented in the work of Pothos and Busemeyer (2013), although without appealing to the operational approach to quantum mechanics. In the series of works of Asano et al. (2010a,b, 2011, 2012), the process of decision making was described by a novel scheme of measurements generalizing the standard theory of quantum apparatuses and instruments (Asano et al., 2010a,b, 2011, 2012).

Now we list once again the main advantageous properties of the quantum instrument/apparatus modeling in cognitive psychology:

1. A possibility to model the feedback reaction of a “mental measurement” (including self-measurements such as decision making and problem judgment) without collapse-like projections of mental states (belief states).
2. The same (self-)measurement output can correspond to a variety of mental state processing.
3. This is the only way to consistently model indirect measurements in which the output of one psychological function of the brain is (self-) measured through the output of another psychological function.

## 3. Quantum states

We start with a brief introduction to the quantum basics and define pure and mixed quantum states. The state space of a quantum system is complex Hilbert space. Denote it by *H*. This is a complex linear space endowed with a scalar product, a positive-definite non-degenerate Hermitian form. Denote the latter by 〈·|·〉. It generates the norm on *H*: $∥\psi ∥=\sqrt{\langle \psi |\psi \rangle }.$

A reader who does not feel comfortable in the abstract framework of functional analysis can simply proceed with the Hilbert space *H* = **C**^*n*^, where **C** is the set of complex numbers, and the scalar product $\langle u|v\rangle =\underset{i}{\sum }u_{i}{\overset{-}{v}}_{i},u=\left(u_{1},\ldots ,u_{n}\right),v=\left(v_{1},\ldots ,v_{n}\right).$ Instead of linear operators, one can consider matrices.

Pure quantum states are represented by normalized vectors, ψ ∈ *H*: ∥ψ∥ = 1. Two colinear vectors, ψ′ = λψ, λ ∈ **C**, |λ| = 1, represent the same pure state. Each pure state can also be represented as the projection operator *P*_ψ_ which projects *H* onto the one dimensional subspace based on ψ. For a vector ϕ ∈ *H*, *P*_ψ_ϕ = 〈ϕ|ψ〉 ψ. Any projector is a Hermitian and positive-definite operator^3^. We also remark that the trace of the one dimensional projector *P*_ψ_ equals to 1: Tr *P*_ψ_ = 1. (We recall that, for a linear operator *A*, its trace can be defined as the sum of diagonal elements of its matrix in any orthonormal basis: $\text{Tr}\;\text{A}=\underset{\text{i}}{\sum }{\text{a}}_{\text{ii}}.$) We summarize these properties of an operator (matrix) ρ = *P*_ψ_ representing a pure state. It is

1. Hermitian,
2. positive-definite,
3. trace one,
4. idempotent: ρ^2^ = ρ.

A linear operator is an orthogonal projector if and only if it satisfies (1) and (4); in particular, (2) is a consequence of (4). The properties (1–4) are characteristic for one dimensional orthogonal projectors—pure states [for a projector, (3) implies that it is one dimensional], i.e., any operator satisfying (1–4) represents a pure state.

The next step in the development of quantum mechanics was the extension of the class of quantum states, from pure states represented by one dimensional projectors to states represented by linear operators (matrices) having the properties (1–3). Such operators (matrices) are called *density operators* (density matrices). (This nontrivial step of extension of the class of quantum states was based on the efforts of Landau and von Neumann). One typically distinguish pure states, as represented by one dimensional projectors, and mixed states, those density operators which cannot be represented by one dimensional projectors. The terminology “mixed” has the following origin: any density operator can be represented as a “mixture” of pure states (ψ_*i*_):

(1) $$\begin{array}{l} \rho =\underset{i}{\sum }p_{i}P_{\psi_{i}},\;p_{i}\in \left[0,1\right],\underset{i}{\sum }p_{i}=1. \end{array}$$

The state is pure if and only if such a mixture is trivial: all *p*_*i*_, besides one, equal to zero. However, by operating with the terminology “mixed state” one has to take into account that the representation in the form Equation (1) is not unique. The same mixed state can be interpreted as mixtures of different collections of pure states.

Any operator ρ satisfying (1–3) is diagonalizable (even in the infinite-dimensional Hilbert space), i.e., in some orthonormal basis it is represented as a diagonal matrix, ρ = diag(p_j_), where $p_{j}\in \left[0,1\right],\underset{j}{\sum }p_{j}=1.$ Thus, it can be represented in the form Equation (1) with mutually orthogonal one dimensional projectors. The property (4) can be used to check whether a state is pure or not. We point out that pure states are merely mathematical abstractions; in real experimental situations it is possible to prepare only mixed states; one defines the degree of purity as Tr[ρ^2^ − ρ]. Experimenters are satisfied by getting this quantity less than some small ϵ.

## 4. Atomic instruments/apparatuses

The notions of instrument and apparatus are based on very simple and natural consideration. Consider systems of any origin (physical, biological, social, financial). Suppose that the states of such systems can be represented by points of some set *X*. These are statistical states, i.e., by knowing the state of a system one can determine the values of observables only with some probabilities. Then, for each state *x* ∈ *X* and observable *A* and its concrete value *a*_*i*_, there is defined a map

(2) $$\begin{array}{l} p_{i}=f_{A,a_{i}}\left(x\right) \end{array}$$

giving the probability of the result *A* = *a*_*i*_ for systems in the state *x* ∈ *X*. Here *f*_*A*,*a*_*i*__ : *X* → [0, 1]. Then its is natural to assume that the measurement modifies the state *x*, i.e., there is is defined another map

(3) $$\begin{array}{l} x_{i}=g_{A,a_{i}}\left(x\right), \end{array}$$

here *g*_*A*,*a*_*i*__ : *X* → *X*. This scheme is applicable both in classical and quantum physics as well as in psychology—Stimulus–Organism–Response (S–O–R) scheme for explaining behavior (Woodworth, 1921) of humans and other cognitive systems.

For the fixed observable *A*, the system of the state transformation maps (*g*_*A*,*a*_*i*__) corresponding to all possible values (*a*_*i*_) of *A* is called an *instrument* and the collection of maps (*f*_*A*,*a*_*i*__; *g*_*A*,*a*_*i*__) is called an *apparatus*. Of course, this scheme is too general and, to get something fruitful, one has to select the state space *X* having a special structure and special classes of *f*- and *g*-maps. Quantum theory is characterized by selection of the state space starting with a complex Hilbert space. This choice leads to theory of *quantum instruments and apparatuses*.

The general theory of quantum measurements is mathematically advanced, Section 9. Therefore, it is useful to illustrate it by a simple example. We consider the simplest class of quantum instruments extending the class of von Neumann–Lüders instruments of the projection type. These are *atomic instruments*.

Suppose that the range of values of a measurement, spectrum of an observable, is discrete *O* = {*a*_1_, …, *a*_*n*_}. The main point of theory of instruments is that each measurement resulting in a concrete value *a*_*i*_ generates the feedback action to the original state ρ of a quantum system, i.e., ρ is transformed into a new state ρ_*a*_*i*__, see Equation (3):

(4) $$\begin{array}{l} \rho \to \rho_{a_{i}}. \end{array}$$

We start with the standard von Neumann–Lüders measurements. which gives us an important class of quantum instruments/apparatuses (especially from the historical viewpoint). These measurements are mathematically represented by Hermitian operators,

(5) $$\begin{array}{l} A=\underset{i}{\sum }a_{i}P_{a_{i}}, \end{array}$$

where *P*_*a*_*i*__ is the projector onto the eigensubspace corresponding to the eigenvalue *a*_*i*_. For pure states, the transformation (Equation 4) is based on the projection *P*_*a*_*i*__:

(6) $$\begin{array}{l} \psi \to P_{a_{i}}\psi , \end{array}$$

this map is linear and it is convenient to work with it. However, if *P*_*a*_*i*__ ≠ *I*, where *I* is the unit operator, then ∥*P*_*a*_*i*__ψ∥ < 1, so the output of Equation (6) is not a state. To get a state, it has to be normalized by its norm:

(7) $$\begin{array}{l} \psi \to \frac{P_{a_{i}}\psi }{∥P_{a_{i}}\psi ∥}. \end{array}$$

This is a map from the space of pure states into the space of pure states, but it is nonlinear. This type of the feedback reaction to the result of measurement was postulated by von Neumann. It is well-known as the *projection postulate* of quantum mechanics (the state reduction postulate or the state collapse postulate, see (Khrennikov and Basieva, 2014) for a psychologist-friendly discussion on these postulates and their role in quantum physics and cognitive psychology and psychophysics) ^4^.

Now, for a pure state ψ, one can consider its representation by the density operator ρ = *P*_ψ_. In such terms, the state transform (Equation 6) can be written as

(8) $$\begin{array}{l} \rho \to P_{a_{i}}\rho P_{a_{i}}. \end{array}$$

This is the simplest example of a transformation which in quantum measurement theory is called a *quantum operation*. It can be extended to the linear map from the space of linear operators (matrices) to itself—by the same formula (Equation 8). For a finite spectral set *O*, the collection of quantum operations (Equation 8), *a*_*i*_ ∈ *O*, gives the simplest example of a *quantum instrument*.

We are again interested in a map from the space of density operators (matrices) to itself, see Equation (4). Thus, we again have to make normalization:

(9) $$\begin{array}{l} \rho \to \rho_{a_{i}}=\frac{P_{a_{i}}\rho P_{a_{i}}}{\text{Tr}\;{\text{P}}_{{\text{a}}_{\text{i}}}\rho {\text{P}}_{{\text{a}}_{\text{i}}}}. \end{array}$$

It is nonlinear and physicists work with quantum operations (forming instruments), by making normalization by trace only at the final step of calculations which can involve a chain of measurements.

However, we are primarily interested not in the measurement feedback to the initial quantum state ρ, but in the probabilities to get the results *a*_*i*_ ∈ *O*. Denote them *p*(*a*_*i*_|ρ). Here they are given by Born's rule. If the initial state is pure ρ = *P*_ψ_, then

(10) $$\begin{array}{l} p\left(a_{i}|\psi \right)=\langle P_{a_{i}}\psi |\psi \rangle =∥P_{a_{i}}\psi {∥}^{2}. \end{array}$$

It is easy to see that

(11) $$\begin{array}{l} p\left(a_{i}|\psi \right)=\text{Tr}\;{\text{P}}_{{\text{a}}_{\text{i}}}{\text{P}}_{\psi }. \end{array}$$

This formula can be easily generalized, e.g., via Equation (1), to an arbitrary initial state ρ:

(12) $$\begin{array}{l} p\left(a_{i}|\rho \right)=\text{Tr}\;{\text{P}}_{{\text{a}}_{\text{i}}}\rho . \end{array}$$

A *quantum apparatus* is the combination of feedback state-transformations, i.e., a quantum instrument, and detection probabilities.

In the von Neumann–Lüders approach the quantum instrument is uniquely determined by an observable, the Hermitian operator *A*. The latter is the basis of the construction. However, even in this approach we could start directly with an instrument determined by a family of mutually orthogonal projectors (*P*_*a*_*i*__), i.e.,

(13) $$\begin{array}{l} \underset{i}{\sum }P_{a_{i}}=I, \end{array}$$

where *P*_*a*_*i*__⊥*P*_*a*_*j*__, *i* ≠ *j*, and then define the observable *A* simply as this family (*P*_*a*_*i*__). In quantum information the values *a*_*i*_ have merely the meaning of *labels* for the results of measurement. For future generalization, we remark that the normalization condition (Equation 13) can be written as

(14) $$\begin{array}{l} \underset{i}{\sum }P_{a_{i}}^{*}P_{a_{i}}=I, \end{array}$$

because, for any orthogonal projector P, P^*^ = *P and P*^2^ = *P*.

Now we move to general atomic instruments and apparatuses. Here quantum operations have the form:

(15) $$\begin{array}{l} \rho \to Q_{a_{i}}\rho Q_{a_{i}}, \end{array}$$

where, for each value *a*_*i*_, *Q*_*a*_*i*__ is a linear operator which is a contraction (i.e., its norm is bounded by 1). These operators are constrained by the normalization condition, cf. (Equation 14):

(16) $$\begin{array}{l} \underset{i}{\sum }Q_{a_{i}}^{*}Q_{a_{i}}=I, \end{array}$$

These operations determine an atomic quantum instrument. Each quantum operation induces the corresponding state transformation:

(17) $$\begin{array}{l} \rho \to \rho_{a_{i}}=\frac{Q_{a_{i}}\rho Q_{a_{i}}}{\text{Tr}\;Q_{{\text{a}}_{\text{i}}}\rho Q_{{\text{a}}_{\text{i}}}}. \end{array}$$

In particular, pure states are transformed into pure states (similar to the von Neumann–Lüders measurements):

(18) $$\begin{array}{l} \psi \to \frac{Q_{a_{i}}\psi }{∥Q_{a_{i}}\psi ∥}. \end{array}$$

Probabilities of the results of measurements are given by the following generalization of Equation (12):

(19) $$\begin{array}{l} p\left(a_{i}|\rho \right)=\text{Tr}\;{\text{M}}_{{\text{a}}_{\text{i}}}\rho , \end{array}$$

where

(20) $$\begin{array}{l} M_{a_{i}}=Q_{a_{i}}^{*}Q_{a_{i}}. \end{array}$$

(We remark that if *Q*_*a*_*i*__ is a projector, then $Q_{a_{i}}^{*}=Q_{a_{i}}$ and $Q_{a_{i}}^{2}=Q_{a_{i}}.$ Thus, in this case (Equation 19) matches with (Equation 12). In this way we obtain the corresponding quantum instrument.

The class of atomic instruments and apparatuses is the most direct generalization of the von Neumann–Lüders class. In particular, in general quantum instruments do not transfer pure states into pure states, see Appendix.

## 5. Bistable perception of Schröder stair

**Figure 1:**
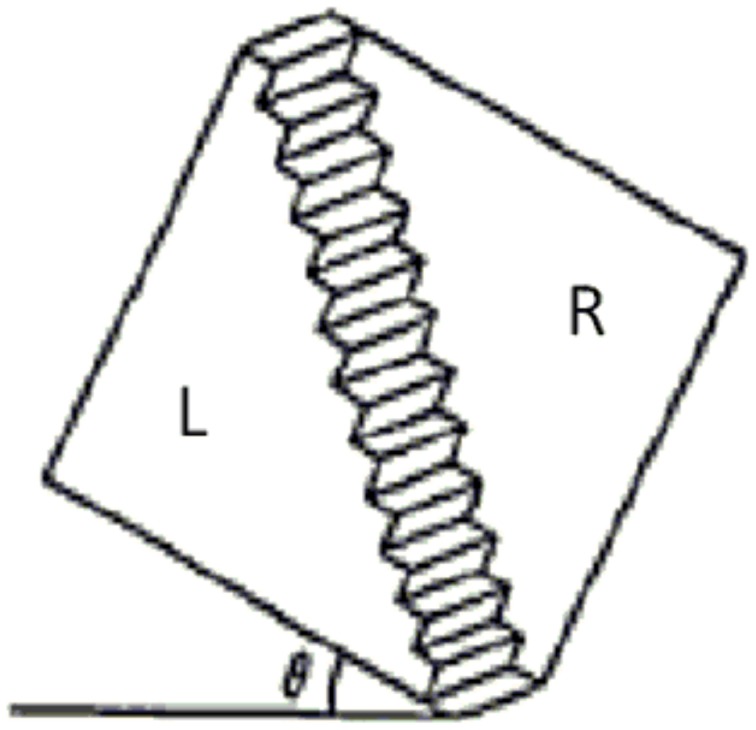
**Schröder Stair is an ambiguous figure which may have two different interpretations, “left part (L) is front and right part (R) is back,” and its converse. Humans percept either of them, and the tendency of the perception depends on the roatating angle θ**.

The experiment is about perception of on the ambiguous figure, the Schröder stair, see Figure 1. Here we reproduce data from paper (Asano et al., 2014), where the reader can find a more detailed presentation.

A total of 151 subjects participated in the test performed at Tokyo University of Science. They were divided into three groups (*n*_*A*_ = 55, *n*_*B*_ = 48, *n*_*C*_ = 48). To the subjects of all three groups, we showed 11 pictures of the Schröder stair which was leaning at different angles. Subjects answered *L* = “I can see that left side is front,”or *R* = “I can see that right side is front” for each picture. Thus, we have a random variable for perception, *X*_θ_ = *L, R*. We denote the experimental probability that a subject answers “Left side is front” by *p*(*X*_θ_ = *L*).

**Figure 2:**
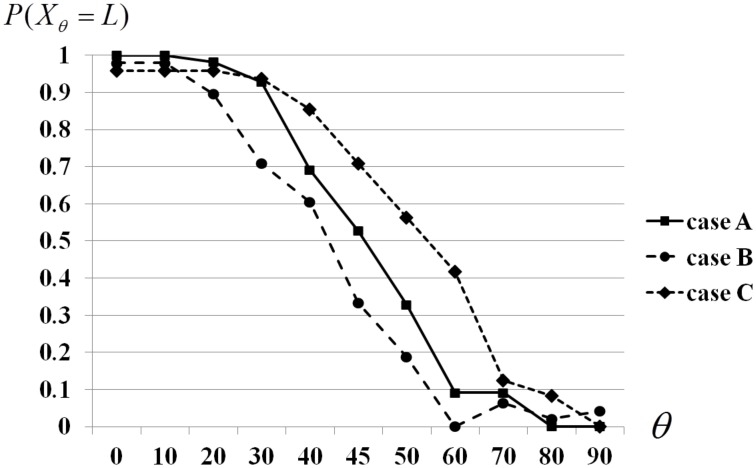
**Optical illusion is affected by memory bias: subject's perception is shifted in response to rotation direction of the figure**.

For the first group (A), order of showing pictures is randomly selected for each subject. For the second group (B), angle θ changed from 0 to 90 as if the picture was rotating clockwise. Inversely, for the third group (C), the angle θ was changed from 90 to 0. As a result, we obtained perception trends with respect of angles, see Figure 2. These graphs demonstrate contextuality of data, its dependence on experimental contexts, (A)–(C), (see Asano et al., 2014) for numerical estimation of the degree of contextuality as violation of the Garg–Leggett inequality. As was discussed in Introduction, contextual statistical data can be modeled by using the quantum formalism.

## 6. Mental apparatuses

We shall proceed with finite dimensional state spaces by making remarks on the corresponding modifications in the infinite dimensional case. The symbol *D*(*H*) denotes the space of density operators in the complex Hilbert space *H*; *L*(*H*) the space of all linear operators in *H* (bounded operators in the infinite dimensional case).

The space *L*(*H*) can itself be endowed with the structure of the linear space. We also have to consider linear operators from *L*(*H*) into itself; such maps, *T* : *L*(*H*) → *L*(*H*) are called *superoperators*. We shall use this notion only in Section 9. Thus, for a moment, the reader can proceed without it.

Moreover, on the space *L*(*H*) it is possible to introduce the structure of Hilbert space with the scalar product

$$\langle A|B\rangle =\text{Tr}\;{\text{A}}^{*}\text{B}.$$

Therefore, for each superoperator *T* : *L*(*H*) → *L*(*H*), there is defined its adjoint (super)operator T^*^ : *L*(*H*) → *L*(*H*), 〈*T*(*A*)|*B*〉 = 〈*A*|T^*^(*B*)〉, *A, B* ∈ *L*(*H*).

For reader's convenience we remind the notion of POVM.

**Definition**. *A positive operator valued measure (POVM) is a family of positive operators* {*M*_*j*_} *such that* $\overset{m}{\underset{j=1}{\sum }}M_{j}=I,$ *where I is the unit operator*.

Consider a cognitive system, to be concrete consider a human individual, call her Keiko. She confronts some recognition-problem, i.e., in our problem of bistable perception of Schröder stair she has to make the choice between two perception *A* = *L, R*. In the quantum(-like) model the space of her mental states is represented by complex Hilbert space *H* (pure states are represented by normalized vectors and mixed states by density operators).

In the model under construction *H* is tensor-factorized into two components, namely, *H* = *H* ⊗ *K*, where *H* is the space of *sensation-states* and *K* is the space of *perception-states*. The states of the latter are open for *conscious introspection*, but the states of the former are in general not approachable consciously. We recall that we model Helmholtz unconscious inference.

In general suppose that Keiko confronts some concrete recognition problem *A* with possible perceptions labeled as *a*_*i*_, *i* = 1, 2, …, *m*. We denote the set of possible values of *A* by the symbol *O*, i.e., *O* = {*a*_1_, …, *a*_*m*_}. By interacting with a figure (in our concrete case the figure is ambiguous) she generates the the sensation-state ρ (e.g., a pure state, i.e., ρ = |ψ〉〈ψ|, ψ ∈ *H*, ∥ψ∥ = 1). The process of generation of ρ can be mathematically represented as a unitary transformation in the space *H*. Denote the pre-recognition state of sensation by ρ_0_. Then

$$\rho =U\rho_{0}U^{*},$$

where the unitary operator *U* : *H* → *H* depends on the figure; in our concrete case *U* = *U*_Schr_.

To come to the concrete perception, Keiko uses a “mental apparatus,” denoted as *A*, which produces the results (perceptions) *a*_*i*_ randomly with the probabilities *p*(*a*_*i*_|ρ), the output probabilities^5^. An apparatus represents not only perceptions and the corresponding probabilities, but also the results of the evolution of the initial sensation-state ρ as induced by the back-reaction to the concrete perception *a*_*i*_. This is a sort of the state reduction, “sensation-state collapse” as the result of creation of the concrete perception *a*_*i*_. Thus, the sensation state ρ which Keiko created from her visual image is transformed into the output state ρ_*a*_*i*__.

However, as we shall see, in general this sensation-state update can be sufficiently peaceful, so our model differs crucially from the orthodox quantum models of cognition (Busemeyer and Bruza, 2012) based on the projection-type state update. Thus, each mental apparatus *A* corresponding to the recognition-problem *A* is mathematically represented by

- probabilities for concrete perceptions *p*(*a*_*i*_|ρ);
- transformations of the initial sensation-state corresponding to the concrete results of perception,
  (21) $$\begin{array}{l} \rho \to \rho_{a_{i}}. \end{array}$$

The rigorous mathematical description of such state transformations leads to the notion of *a quantum instrument*, see Section 9.

### 6.1. Mixing law

In the quantum operational formalism it is assumed that these probabilities, *p*(*a*_*i*_|ρ), satisfy the *mixing law*. We remark that, for any pair of states (density operators) ρ_1_, ρ_2_ and any pair of probability weights *q*_1_, *q*_2_ ≥ 0, *q*_1_+*q*_2_ = 1, the convex combination ρ = *q*_1_ρ_1_+*q*_2_ρ_2_ is again a state (density operator). In accordance with the mixing law any apparatus produces probabilities such that

(22) $$\begin{array}{l} p\left(a_{i}|q_{1}\rho_{1}+q_{2}\rho_{2}\right)=q_{1}p\left(a_{i}|\rho_{1}\right)+q_{2}p\left(a_{i}|\rho_{2}\right). \end{array}$$

In our model of bistable perception the mixing law can be formulated as follows:

A probabilistic mixture of sensations produces the mixture of probabilities for perception outputs.

In physics this is a very natural assumption. However, in modeling of cognitive phenomena, in particular, unconscious inference, an additional analysis of its validity has to be performed. We have no possibility to do this in this note, so we postpone such analysis to one of coming publications. Now we mimic quantum physics explicitly and proceed under the assumption (Equation 22).

### 6.2. Composition of the apparatuses

It is natural to assume that after resolving the recognition-problem *A* a person is ready to look at another image *B* and proceed to its perception. In general perception of *B* depends on the preceding perception of *A*. Such a sequence of perceptions represented as a new mental apparatus, the composition of the apparatuses *A* and *B* : *BA*. Its outputs are ordered pairs of perceptions (*a*_*i*_, *b*_*j*_). It is postulated that the corresponding output probabilities and states are determined as

(23) $$\begin{array}{rcl} p\left(\left(a_{i},b_{j}\right)|\rho \right) & = & p\left(b_{j}|\rho_{a_{i}}\right)p\left(a_{i}|\rho \right); \end{array}$$

(24) $$\begin{array}{rcl} \rho_{\left(a_{i},b_{j}\right)} & = & {\left(\rho_{a_{i}}\right)}_{b_{j}}. \end{array}$$

The law (Equation 23) can be considered as the quantum generalization of the Bayes rule. The law (Equation 24) is the natural composition law.

In our experiment with rotation of the Schröder stair, we are interested in a sequence of instruments *A*_θ_ corresponding to some sample of angles *C* = {θ_1_, …, θ_*m*_}. Here *C* determines the context of the experiment. Our data from Section 5 can be represented as the superposition of quantum apparatuses: *A*_*C*_ = *A*_θ_*m*__…*A*_θ_1__. Here *A*_*C*_ is the quantum apparatus representing the context *C*. In our experimental study we considered not only deterministic contexts corresponding to clockwise and counter-clockwise rotations, but even the random context determined by the uniform probability distribution.

## 7. Perception through unitary interaction between the sensation and perception-states

The above operational description of “perception–production” was formulated solely in terms of sensation-states. However, a sensation-state is a complex informational state which is in general unapproachable for conscious introspective. The operational representation of observables in the space of sensation-states is not straightforward and in general it cannot be formulated in terms of mutually exclusive perceptions. For example, in our experiment Keiko's perceptions can be binary encoded: *A* = *L, R*. However, her sensation of the Schröder stair is a complex information state depending on a variety of parameters (in particular, we are interested in dependence on the rotation angle). *The subspaces corresponding to sensations leading to the L-perception and R-perception are in general not orthogonal*. This non-orthogonality of sensation subspaces for different perceptions is the fundamental feature of bistable perception, recognition of ambiguous figures.

Therefore, it is more fruitful to define the perception-observable directly by using an additional state space, the space of the perception-states *K*. *In the perception space a perception-observable can be defined as the standard von Neumann–Lüders projection observable.*

**Example 1**. Consider the simplest case: recognition of the fixed figure *A*, with dichotomous output, i.e., there are two possible outcomes of “perception-measurement,” e.g., *L* = 0 and *R* = 1 for the Schröder stair. This observable can be represented by the pair of projectors (*P*_0_, *P*_1_) onto the subspaces *K*_0_ and *K*_1_ of the perception space *K*. Since the perceptions *a*_0_ = 0 and *a*_1_ = 1 are mutually exclusive, and sharply exclusive, the subspaces *K*_0_ and *K*_1_ are orthogonal. Hence, the projectors *P*_0_ and *P*_1_ can be selected as orthogonal. The perception-observable *A* can be represented as the conventional von-Neumann-Lüders observable Â = *a*_0_*P*_0_+*a*_1_*P*_1_(= *P*_1_). However, we emphasize that this representation is valid only in the perception-state space *K*. It is often (but not always!) possible to proceed with one dimensional projectors, i.e., to represent possible perceptions just by the basis vectors in the two dimensional perception-state space, (|0〉, |1〉). Here each perception-state can be represented as superposition

(25) $$\begin{array}{l} \phi =c_{0}|0\rangle +c_{1}|1\rangle ,\;|c_{0}{|}^{2}+|c_{1}{|}^{2}=1. \end{array}$$

Measurement of *A* leads to probabilities of perceptions given by squared coefficients, $p_{0}=|c_{0}{|}^{2},p_{1}=|c_{1}{|}^{2}.$

In the case of the finite-dimensional perception-state, a perception-observable *A* can be represented as

(26) $$\begin{array}{l} A=\underset{i}{\sum }a_{i}P_{i}, \end{array}$$

where (*P*_*i*_) is the family of mutually orthogonal projectors in the space of perception-states *K* and (*a*_*i*_) are real numbers encoding possible answers (perceptions).

Now we shall explore the cognitive analog of the standard scheme of *quantum indirect measurements.*

In our cognitive framework “indirectness” means that the sensation-states are in general unapproachable for consicious introspection. Therefore, it is impossible to perform the direct measurement on the sensation-state ρ (in particular, on a pure state ρ = |ψ〉〈ψ|). Moreover, in the sensation-state the alternatives, say 0/1, encoded in a perception-observer *A* are not represented exclusively, they can have overlap. (Mathematically the overlap is expressed as non-orthogonality of sensation-subspaces corresponding to various perceptions.)

In the quantum measurement framework, this situation is described as follows: in the sensation space an observable *A* is represented as an unsharp observable of the POVM-type. Roughly speaking in the *H*-representation the *A*-zero contains partially the *A*-one and vice versa. The latter is simply a consequence of interpretation of POVM observables as unsharp observables.

**Remark 1**. To map the quantum physics scheme (Ozawa, 1997) of indirect measurements onto the quantum(-like) cognition scheme, one has to associate the state of the principle physical system *S* with the sensation-state and the state of the probe physical system *S*′ with the perception-state. We point out that in the cognitive framework we do not consider analogs of physical systems. In principle, one can consider the sensation-system *S* as a part of the neuronal system representing sensations and the perception system *S*′ as another part of the neuronal system representing possible perceptions. The latter can be specified: different measurements can be associated with different neuronal networks responsible for the corresponding perceptions. However, in principle we need not associate sensation and perception states with the concrete physical neuronal networks. In the case of cognition usage of isolated physical systems as carriers of the corresponding information states might be ambiguous. The interconnectivity of neuronal networks is very high. Therefore, the picture of distributed computational system is more adequate. (Of course, even in physics the notion of an isolated system is just an idealization of the real situation). Therefore, it is useful to proceed in the *purely information approach* by operating solely with states, without coupling them to bio-physical systems. This is, in fact, the quantum information approach, where systems play the secondary role, and one operates with states; especially for the information interpretation of quantum mechanics (Zeilinger, 2010).

In the simplest model we can assume that at the beginning of the process of perception-creation the sensation and perception-states, ρ and σ, are not entangled^6^. Thus, mathematically, in accordance with the quantum formalism, the integral sensation–perception-state, the complete mental state corresponding to the problem under consideration, can be represented as the tensor product

$$\begin{array}{l} R=\rho ⊗\sigma . \end{array}$$

In the process of perception-creation the sensation and perception-states (cf. Remark 1) “interacts” and the evolution of the sensation–perception-state *R* is mathematically represented by a unitary operator^7^ *U* : *H* → *H*:

(27) $$\begin{array}{l} R\to R_{\text{out}}\equiv URU^{*}. \end{array}$$

In the space of sensation–perception-states *H* the perception-observer *A* is represented by the operator *I* ⊗ *A*. Thus, the probabilities of perceptions are given by

(28) $$\begin{array}{l} p_{a_{i}}^{A⊗I}=\text{Tr}\;{\text{R}}_{\text{out}}\left(\text{I}⊗{\text{P}}_{\text{i}}\right)=\text{Tr}\;\text{UR}{\text{U}}^{*}\left(\text{I}⊗{\text{P}}_{\text{i}}\right), \end{array}$$

where the projectors (*P*_*i*_) form the spectral decomposition of the Hermitian observable *A* in *K*, see Equation (26).

Since only the perception-state belonging *K* is a subject of conscious introspective, at the conscious level the perception process can be represented solely in the state space *K*. The post-interaction perception-state σ_out_ can be (mathematically) extracted from the integral state *R*_out_ with the aid of the operation of the partial trace:

(29) $$\begin{array}{l} \sigma_{\text{out}}={\text{Tr}}_{\text{H}}{\text{R}}_{\text{out}}. \end{array}$$

Then perceptions can be represented as the results of the *A*-measurement (measurement of the projection-type) in the perception space; measurement on the output state σ_out_. The probabilities of the concrete perceptions (*a*_*i*_) are given by the standard Born rule:

(30) $$\begin{array}{l} p_{a_{i}}^{A}={\text{Tr}}_{\text{K}}\sigma_{\text{out}}{\text{P}}_{\text{i}}={\text{Tr}}_{\text{K}}\left({\text{Tr}}_{\text{H}}{\text{R}}_{\text{out}}\right){\text{P}}_{\text{i}}=\text{Tr}{\text{R}}_{\text{out}}\left(\text{I}⊗{\text{P}}_{\text{i}}\right)={\text{p}}_{{\text{a}}_{\text{i}}}^{\text{A}⊗\text{I}}. \end{array}$$

Thus, Equations (28) and (30) match each other.

If the concrete result *A* = *a*_*i*_ was observed, then the state of perception σ is transformed into

(31) $$\begin{array}{l} \sigma_{i;\text{out}}=\frac{{\text{Tr}}_{\text{H}}{\text{R}}_{\text{out}}\left(\text{I}⊗{\text{P}}_{\text{i}}\right)}{\text{Tr}{\text{R}}_{\text{out}}\left(\text{I}⊗{\text{P}}_{\text{i}}\right)}. \end{array}$$

What does happen in the sensation space?

The expression (Equation 28) for the probability of the perception *a*_*i*_ can be represented as

(32) $$\begin{array}{l} p\left(a_{i}|\rho \right)=p_{a_{i}}^{A⊗I}=\text{Tr}{\text{R}}_{\text{out}}\left(\text{I}⊗{\text{P}}_{\text{i}}\right)=\text{Tr}\rho ⊗\sigma {\text{U}}^{*}\left(\text{I}⊗{\text{P}}_{\text{i}}\right)\text{U}\\ ={\text{Tr}}_{\text{H}}\rho {\text{M}}_{{\text{a}}_{\text{i}}}, \end{array}$$

where

(33) $$\begin{array}{l} M_{a_{i}}={\text{Tr}}_{\text{K}}\left(\text{I}⊗\sigma \right){\text{U}}^{*}\left(\text{I}⊗{\text{P}}_{\text{i}}\right)\text{U}. \end{array}$$

The operator *M*_*i*_; *H* → *H* can also be represented in the following useful form (a consequence of the cyclic property of the trace operation):

(34) $$\begin{array}{l} M_{a_{i}}={\text{Tr}}_{\text{K}}{\text{U}}^{*}\left(\text{I}⊗{\text{P}}_{\text{i}}\right)\text{U}\left(\text{I}⊗\sigma \right) \end{array}$$

We remark that (Equation 33) implies:

$$\begin{array}{l} \underset{i}{\sum }M_{a_{i}}={\text{Tr}}_{\text{K}}\left(\text{I}⊗\sigma \right){\text{U}}^{*}\left(\text{I}⊗\underset{\text{i}}{\sum }{\text{P}}_{\text{i}}\right)\text{U}={\text{Tr}}_{\text{K}}\text{I}⊗\sigma =\left({\text{Tr}}_{\text{K}}\sigma \right)\text{I}. \end{array}$$

We also remark that each operator *M*_*a*_*i*__ is positively defined and Hermitian.

Thus, in the sensation space the perception-observable of the projection-type *A* (acting in *K*) with the spectral family (*P*_*i*_) is represented as POVM *M* = (*M*_*i*_). We remark that in general the operators *M*_*i*_ are not projectors. Such measurement cannot separate sharply sensations leading to perceptions (*a*_*i*_) for different *i*.

The operational formalism also gives the “post-perception sensation-state,” i.e., the state of sensation created as the feedback to the consciously recognized perception *a*_*i*_,

(35) $$\begin{array}{l} \rho_{a_{i}}=\frac{{\text{Tr}}_{\text{K}}{\text{R}}_{\text{out}}\left(\text{I}⊗{\text{P}}_{\text{i}}\right)}{\text{Tr}{\text{R}}_{\text{out}}\left(\text{I}⊗{\text{P}}_{\text{i}}\right)}. \end{array}$$

The output sensation-state depends not only on the initial sensation-state ρ, but also on the initial perception-state σ, interaction between believes and possible perceptions given by *U* and the question-observable *A* acting in *K*.

## 8. The indirect measurement scheme for rotation contexts for perception of Schröder stair

As at the very end of Section 6.2, we consider contextual measurements for the Schröder stair: a sequence of perceptions corresponding to some sample of angles *C* = {θ_1_, …, θ_*m*_}. Here *C* determines the context of the experiment. We apply the scheme of indirect measurements. We can assume that the perception space *K* is two dimensional with the orthogonal basis |*L*〉, |*R*〉 representing the “left-faced” and “right-faced” preceptions of the stair. Thus, projectors *P*_*i*_, *i* = *L, R*, are one dimensional.

We start with the initial sensation state ρ_0_. By the visual image rotated at the angle θ_1_ this state is transformed to

(36) $$\begin{array}{l} \rho_{\theta_{1}}=U_{\text{Sch};\theta_{1}}\rho_{0}U_{\text{Sch};\theta_{1}}^{*}, \end{array}$$

where *U*_Sch;θ_1__ represents the unitary dynamics induced by this image. Then the perception of the image is modeled starting with

(37) $$\begin{array}{l} R_{\theta_{1}}=\rho_{\theta_{1}}⊗\sigma_{0}, \end{array}$$

where σ_0_ represents the state of perception preceding interaction with the state of sensation. It is natural to assume that σ_0_ = |ϕ_0_〉〈ϕ_0_|, where

(38) $$\begin{array}{l} \phi_{0}=\left(|L\rangle +|R\rangle \right)∕\sqrt{2} \end{array}$$

is the neutral composition of the states “left-faced” and “right-faced.” It represents the deepest state of uncertainty. Suppose (for simplicity) that independently of the angle the interaction of sensation and perception states is given by the same unitary operator *U*. Then Keiko's perception of the Schröder stair observed at the angle θ_1_ with the fixed result *i*_1_ = *L* or *R* leads to the new states of sensation and perception:

(39) $$\begin{array}{l} \sigma_{i_{1};\theta_{1}}=\frac{{\text{Tr}}_{\text{H}}\text{U}{\text{R}}_{\theta_{1}}{\text{U}}^{*}\left(\text{I}⊗{\text{P}}_{{\text{i}}_{1}}\right)}{\text{TrU}{\text{R}}_{\theta_{1}}{\text{U}}^{*}\left(\text{I}⊗{\text{P}}_{{\text{i}}_{1}}\right)},\;\rho_{i_{1};\theta_{1}}=\frac{{\text{Tr}}_{\text{K}}\text{U}{\text{R}}_{\theta_{1}}{\text{U}}^{*}\left(\text{I}⊗{\text{P}}_{{\text{i}}_{1}}\right)}{\text{TrU}{\text{R}}_{\theta_{1}}{\text{U}}^{*}\left(\text{I}⊗{\text{P}}_{{\text{i}}_{1}}\right)}. \end{array}$$

The probability of creation of the perception *i* can be calculated as

(40) $$\begin{array}{l} p_{i_{1};\theta_{1}}={\text{Tr}}_{\text{H}}\rho_{\theta_{1}}{\text{M}}_{{\text{i}}_{1};\theta_{1}}. \end{array}$$

Here POVM's component *M*_*i*_1_; θ_1__, *i*_1_ = *L, R*, has the form:

(41) $$\begin{array}{l} M_{i_{1};\theta_{1}}={\text{Tr}}_{\text{K}}{\text{U}}^{*}\left(\text{I}⊗{\text{P}}_{{\text{i}}_{1}}\right)\text{U}\left(\text{I}⊗\sigma_{0}\right). \end{array}$$

For the next measurement corresponding to rotation of Schröder's stair for the angle θ_2_, Keiko selects ρ_*i*_1_; θ_1__ and σ_*i*_2_; θ_1__ as the initial states. This means that creation of the fixed perception *i*_1_ leads to disentanglement of her mental state into the product of two states, the state of sensation and perception. Then

(42) $$\begin{array}{l} \rho_{\theta_{2}}=U_{\text{Sch};\theta_{2}}\rho_{i_{1};\theta_{1}}U_{\text{Sch};\theta_{2}}^{*}, \end{array}$$

where *U*_Sch;θ_1__ represents the unitary dynamics induced by the θ_2_-image. Then

(43) $$\begin{array}{l} R_{\theta_{2}}=\rho_{\theta_{2}}⊗\sigma_{i_{1};\theta_{1}}, \end{array}$$

Then Keiko's perception of the Schröder stair observed at the angle θ_2_ with the fixed result *j* = *L* or *R* leads to the new states of sensation and perception:

(44) $$\begin{array}{l} \sigma_{i_{2};\theta_{2}}=\frac{{\text{Tr}}_{\text{H}}\text{U}{\text{R}}_{\theta_{2}}{\text{U}}^{*}\left(\text{I}⊗{\text{P}}_{{\text{i}}_{2}}\right)}{\text{TrU}{\text{R}}_{\theta_{2}}{\text{U}}^{*}\left(\text{I}⊗{\text{P}}_{{\text{i}}_{2}}\right)},\;\rho_{i_{2};\theta_{2}}=\frac{{\text{Tr}}_{\text{K}}\text{U}{\text{R}}_{\theta_{2}}{\text{U}}^{*}\left(\text{I}⊗{\text{P}}_{{\text{i}}_{2}}\right)}{\text{TrU}{\text{R}}_{\theta_{2}}{\text{U}}^{*}\left(\text{I}⊗{\text{P}}_{{\text{i}}_{2}}\right)}. \end{array}$$

The probability of creation of the perception *i*_2_ can be calculated as

(45) $$\begin{array}{l} p_{i_{2};\theta_{2}}={\text{Tr}}_{\text{H}}\rho_{\theta_{2}}{\text{M}}_{{\text{i}}_{2};\theta_{2}}. \end{array}$$

Starting with ρ_*i*_2_; θ_2__, σ_*i*_2_; θ_2__, Keiko generates the perception of the θ_3_-rotated stair and so on. After the last test, Keiko's states of sensation and perception ρ_*i*_*n*_; θ_*n*__, σ_*i*_*n*_; θ_*n*__ depend on the sequence of angles *C* and the sequence of her perceptions (*i*_1_, *i*_2_, …, *i*_*n*_). The same is valid for the probability *p*_*i*_*n*_; θ_*n*__. If the experiment is performed for two different contexts *C* = {θ_1_, …, θ_*m*_} and $C^{\prime }=\left\{\theta_{1}^{\prime },\ldots ,\theta_{m}^{\prime }\right\}.$ Then in general it is impossible to embed the probabilities of perceptions in a single Kolmogorov probability space. Therefore, the use of quantum theory of measurement and “quantum probabilities” can be fruitful. Our approach provides the possibility to model probabilities of perceptions depending on a context, a sequence of angles.

## 9. Representing perception by quantum instruments

The considered model of perception as the result of unitary interaction between the sensation-state and the perception-state describes an important class of transformations of the sensation-state, see Equation (35). We now turn to the general case which was considered in Section 6, see Equation (21). Set

(46) $$\begin{array}{l} E\left(a_{i}\right)\rho =p\left(a_{i}|\rho \right)\rho_{a_{i}} \end{array}$$

and, for a subset Γ of *O*, where *O* = {*a*_1_, …, *a*_*m*_} is the set of all possible perceptions, we set

(47) $$\begin{array}{l} E\left(\Gamma \right)\rho =\underset{a_{i}\in \Gamma }{\sum }E\left(a_{i}\right)\rho =\underset{a_{i}\in \Gamma }{\sum }p\left(a_{i}|\rho \right)\rho_{a_{i}}. \end{array}$$

We point to the basic feature of this map:

(48) $$\begin{array}{l} \text{TrE}\left(\text{O}\right)\rho =\underset{{\text{a}}_{\text{i}}\in \text{O}}{\sum }\text{p}\left({\text{a}}_{\text{i}}|\rho \right)\text{Tr}\rho_{{\text{a}}_{\text{i}}}=1. \end{array}$$

For each concrete perception *a*_*i*_, *E*(*a*_*i*_) maps density operators to linear operators (in the infinite dimensional case, these are trace-class operators, but we proceed in the finite dimensional case, where all operators have finite traces).

The mixing law implies that, for any Γ ⊂ *O*,

(49) $$\begin{array}{l} E\left(\Gamma \right)\left(q_{1}\rho_{1}+q_{2}\rho_{2}\right)=q_{1}E\left(\Gamma \right)\rho_{1}+q_{2}E\left(\Gamma \right)\rho_{2}. \end{array}$$

As was shown by Ozawa (1997), under the assumption on the existence of composition of the apparatuses any such a map *E*(Γ) : *D*(*H*) → *L*(*H*) can be extended to a linear map (superoperator)

(50) $$\begin{array}{l} E\left(\Gamma \right):L\left(H\right)\to L\left(H\right) \end{array}$$

such that:

- each *E*(Γ) is positive, i.e., it transfers the set of positively defined operators into itself;
- $E\left(O\right)=\underset{i}{\sum }E\left(a_{i}\right)$ is trace preserving:
  (51) $$\begin{array}{l} \text{TrE}\left(\text{O}\right)\rho =\text{Tr}\rho . \end{array}$$

The latter property is a consequence of Equation (48)^8^.

Thus, the two very natural and simple assumptions, the mixing law for probabilities and the existence of composite apparatuses, have the fundamental mathematical consequence, the representation of the evolution of the state by a superoperator (Equation 50).

In quantum physics such maps are known as state transformers (Busch et al., 1995) or DL (Davis–Levis, Davies and Lewis, 1970) quantum operations^9^.

Thus, each perception induces the back-reaction which can be formally represented as a state transformer. In these terms

(52) $$\begin{array}{l} \rho_{a_{i}}=\frac{E\left(a_{i}\right)\rho }{\text{TrE}\left({\text{a}}_{\text{i}}\right)\rho } \end{array}$$

We remark that the map Γ → *L*(*L*(*H*)), from subsets of the set of possible perceptions *O* into the space of superoperators, is additive:

(53) $$\begin{array}{l} E\left(\Gamma_{1}\cup \Gamma_{2}\right)=E\left(\Gamma_{1}\right)+E\left(\Gamma_{2}\right),\;\Gamma_{1}\cap \Gamma_{2}=\emptyset . \end{array}$$

This is a measure with values in the space *L*(*L*(*H*)). Such measures are called (DL) instruments (Davies and Lewis, 1970). To specify the domain of applications in our case, we shall call them *perception instruments.*

The class of such instruments is essentially wider than the class of instruments based on the unitary interaction between sensation and perception components of the mental state, see Equation (35). The evident generalization of the scheme of Section 7 is to consider nonunitary interactions between the components of the mental state; another assumption which can be evidently violated in modeling of cognition is that the initial sensation and perception states are not entangled (“independent”) (see Asano et al., 2010a,b, 2011, 2012) for generalizations of the aforementioned scheme.

We start with a discussion on possible *nonunitarity of interaction between the sensation and perception states*. In quantum physics the assumption of unitarity of interaction between the principle system *S* and the probe system *S*′ (representing a part of the measurement apparatus interacting with *S*) is justified, because the compound system $S+{\tilde{S}}^{\prime }$ can be considered (with a high degree of approximation) as an isolated quantum system and its evolution can be described (at least approximately) by the Schrödinger equation. And the latter induces the unitary evolution of a state.

In cognition the situation is totally different. The main scene of cognition is not the physical space-time, but the brain. It is characterized by huge interconnectivity and parallelism of information processing. Therefore, it is more natural to consider the sensation and perception states corresponding to different visual inputs as interacting, especially at the level of the sensation-states. Thus, the perception-creation model based on the assumption of isolation of different perception-creation processes from each other seems to be too idealized, although it can be used in many applications, where the concentration on one fixed problem may diminish the influence of other perception-creation processes.

In physics, the assumption that the initial state of the system $S+{\tilde{S}}^{\prime }$ is factorized is also justified, since the exclusion of the influence of the state of the measurement device to the state of a system *S* prepared for measurement (and vice versa) is the experimental routine. In cognition the situation is more complicated. One cannot exclude that in some situations *the initial sensation and perception state are entangled.*

The representation of probabilities with the aid of POVMs is not a feature of only the unitary interaction representation of apparatuses, see Equation (32). In general, any DL-instrument generates such a representation. Take an instrument *E*, where, for each *a*_*i*_ ∈ *O, E*(*a*_*i*_):*L*(*H*) → *L*(*H*) is a superoperator. Then we can define the adjoint operator $E^{*}\left(a_{i}\right):L\left(H\right)\to L\left(H\right).$ Set $M_{a_{i}}=E^{*}\left(a_{i}\right)I,$ where *I* : *H* → *H* is the unit operator. Then, since $p_{a_{i}}=\text{TrE}\left({\text{a}}_{\text{i}}\right)\rho =\text{Tr}\;\text{I};\text{E}\left({\text{a}}_{\text{i}}\right)\rho =\langle \text{I}|\text{E}\left({\text{a}}_{\text{i}}\right)\rho \rangle ==\langle {\text{E}}^{*}\left({\text{a}}_{\text{i}}\right)\text{I}|\rho \rangle =\text{Tr}\left({\text{E}}^{*}\left({\text{a}}_{\text{i}}\right)\text{I}\right)\rho =\text{Tr}{\text{M}}_{{\text{a}}_{\text{i}}}\rho .$ By using the properties of an instrument it is easy to show that *M*_*a*_*i*__ is POVM. Thus, each mental apparatus can be represented by a POVM. We interpret this POVM as the mathematical representation of “unconscious" inference. Such “unconscious measurements” are not sharp, they cannot separate completely different perceptions *a*_*i*_ which are mutually exclusive at the conscious level. Mathematically, we have that the subspaces *H*_*a*_*i*__ = *M*_*i*_*H* need not be orthogonal. Sensation states corresponding to the perceptions *a*_*i*_ and *a*_*j*_, say ψ_*i*_ ∈ *H*_*a*_*i*__ and ψ_*j*_ ∈ *H*_*a*_*j*__, in general have nonzero overlap 〈ψ_*i*_|ψ_*j*_〉 ≠ 0.

## 10. Concluding remarks

This paper is an attempt to present the theory of generalized quantum measurements based on quantum apparatuses and instruments in a humanities-friendly way. This is a difficult task, since this theory is based on advanced mathematical apparatus. We hope that the reader can at least follow our introductory presentation in Sections 3, 4. Although we applied quantum apparatuses and instruments to the concrete problem of cognition, modeling bistable perception and, more generally, Helmholtz unconscious inference, this approach can be used to model general unconscious–conscious information processing. We hope that in future other interesting examples will be presented with the aid of this formalism (cf. Khrennikov, 2010a, 2014).

### Conflict of interest statement

The authors declare that the research was conducted in the absence of any commercial or financial relationships that could be construed as a potential conflict of interest.

## Footnotes

^1^Unconscious inference (Conclusion) is a term of perceptual psychology invented by von Helmholtz (1866); Boring (1942), to describe an involuntary, pre-rational and reflex-like mechanism which is part of the formation of visual impressions.

^2^We remark that the formula of total probability and the Bell-type inequalities can be treated as just two special statistical tests of non-classicality of the data (see Conte et al., 2008; Bruza et al., 2010; Khrennikov, 2010b; Asano et al., 2014; Dzhafarov and Kujala, 2014) for discussion. This is the “minimal interpretation.” In quantum physics the standard interpretation of these inequalities is related to whether we can proceed with a realistic and local model. The Garg–Leggett inequality is a rather special type of Bell's inequalities, since it is about time correlations for a single system and the original Bell's inequality is about spatial correlations for pairs of systems.

^3^We recall that a linear operator *A* in *H* is called Hermitian if it coincides with its adjoint operator, *A* = *A*^*^. If an orthonormal basis in *H* is fixed, (*e*_*i*_), and *A* is represented by its matrix, *A* = (*a*_*ij*_), where *a*_*ij*_ = 〈*Ae*_*i*_|*e*_*j*_〉, then it is Hermitian if and only if ā_*ij*_ = *a*_*ji*_. A linear operator is positive-definite if, for any ϕ ∈ *H*, 〈*A*ϕ|ϕ〉 ≥ 0. It is equivalent to positive definiteness of its matrix. We remark that, for a Hermitian operator, all its eigenvalues are real.

^4^It is less known (in fact, practically unknown) that von Neumann sharply distinguished the case of observables with non-degenerate spectra, i.e., all (*P*_*a*_*i*__) in the spectral decomposition of *A*, see Equation (5), are one dimensional projectors, and degenerate spectra, i.e., some of (*P*_*a*_*i*__) are projectors onto multi-dimensional subspaces. In the first case he postulated aforementioned state-collapse (Equation 7), but in the second case he pointed out that the measurement feedback can generate state transformations different from one given by Equation (7); in particular, the output of the initial pure state can be a mixed state. Later Lüders extended the von Neumann projection postulate even to projectors with degenerate spectra, i.e., in fact, he reduced the class of possible state transformations (quantum operations). This simplification was convenient in theoretical studies and the projection postulate was widely treated as applicable generally, i.e., even to observables with degenerate spectra. The name of Lüders was washed out from the majority of foundational works and nowadays the projection postulate is typically known as the von Neumann projection postulate (see Khrennikov, 2008) for more details.

^5^We are going toward creation of a cognitive analog of the quantum operational model of measurements with the aid of physical apparatuses.

^6^One can say that they are independent. But one can use this terminology carefully, since the notion of quantum independence is more complicated than the classical one and it is characterized by diversity of approaches.

^7^As was mentioned, in the works of Asano et al. (2010a,b, 2011, 2012) and Accardi et al. (in press) even non-unitary evolutions were in charge.

^8^If one wants to extend *E*(Γ) from the set of density operators to the set of all linear operators (in the infinite dimensional case it has to be the set of finite-trace operators) by linearity then it has to be set *E*(Γ)μ = *E*(Γ)Trμ(μ∕Trμ) = Trμ E(Γ)(μ∕Trμ) and, in particular, *E*(*O*)μ = Trμ E(*O*)(μ∕Trμ) = Trμ.

^9^DL-notion of the quantum operation is more general than the notion used nowadays. The latter is based on complete positivity, instead of simply positivity as the DL-notion, see Appendix for the corresponding definition and a discussions on whether the reasons used in physics to restrict the class of state transformers can be automatically used in cognitive science.

## Appendix

### Do we need complete positivity?

Nowadays theory of the DL-instruments is considered old-fashioned; the class of such instruments is considered to be too general: it contains mathematical artifacts which have no relation to real physical measurements and state transformations as back-reactions to these measurements. The modern theory of instruments is based on *the extendability postulate* (e.g., Busch et al., 1995; Ozawa, 1997; Nielsen and Chuang, 2000):

For any apparatus *A*_*S*_ corresponding to measurement of observable *A* on a system *S* and any system $\tilde{S}$ noninteracting with *S* there exists an apparatus $A_{S+\tilde{S}}$ representing measurement on the compound system $S+\tilde{S}$ such that

- *p*(*a*_*i*_|ρ ⊗ *r*) = *p*(*a*_*i*_|ρ);
- (ρ ⊗ *r*)_*a*_*i*__ = ρ_*a*_*i*__ ⊗ *r*

for any state ρ of *S* and any state *r* of $\tilde{S}.$

In physics this postulate is quite natural: if, besides the quantum system *S* which is the object of measurement, there is (somewhere in the universe) another system $\tilde{S}$ which is not entangled with *S*, i.e., their joint pre-measurement state has the form ρ ⊗ *r*, then the measurement on *S* with the result *a*_*i*_ can be considered as measurement on $S+\tilde{S}$ as well with the same result *a*_*i*_. It is clear that the back-reaction cannot change the state of $\tilde{S}.$ Surprisingly this very trivial assumption has tremendous mathematical implications.

Since we proceed only in the finite dimensional case, the corresponding mathematical considerations are simplified. Consider an instrument *E*_*S*_ representing the state update as the result of the back-reaction from measurement on *S*. For each Γ, this is a linear map from *L*(*H*) → *L*(*H*), where *H* is the state space of *S*. Let *W* be the state space of the system $\tilde{S}.$ Then the state space of the compound system $S+\tilde{S}$ is given by the tensor product *H* ⊗ *W*. We remark that the space of linear operators in this state space can be represented as *L*(*H* ⊗ *W*) = *L*(*H*) ⊗ *L*(*W*). Then the superoperator *E*_*S*_(Γ) : *L*(*H*) → *L*(*H*) can be trivially extended to the superoperator *E*_*S*_(Γ) ⊗ *I* : *L*(*H* ⊗ *W*) → *L*(*H* ⊗ *W*). It is easy to prove that the state transformer corresponding to the apparatus for measurements on $S+\tilde{S}$ has to have this form $E_{S+\tilde{S}}\left(a_{i}\right)=E_{S}\left(a_{i}\right)⊗I.$ Hence, this operator also has to be positively defined. We remark that if the state space *W* has the dimension *k*, then the space of linear operators *L*(*W*) can be represented as the space of *k* × *k* matrices which is further denoted as **C**^*k*×*k*^.

Formally, a superoperator *T* : *L*(*H*) → *L*(*H*) is called *completely positive* if it is positive and each its trivial extension *T* ⊗ *I* : *L*(*H*) ⊗ **C**^*k*×*k*^ → *L*(*H*) ⊗ **C**^*k*×*k*^ is also positive. There are natural examples of positive maps which are not completely positive (Nielsen and Chuang, 2000).

A CP quantum operation is a DL quantum operation which is additionally completely positive; a CP instrument is based on CP quantum operations representing back-reactions to measurement. As was pointed out, in modern literature only CP quantum operations and instruments are in the use, so they are called simply quantum operations and instruments.

The main mathematical feature of (CP) quantum operations is that the class of such operations can be described in a simple way, namely, with the aid of the Kraus representation (Busch et al., 1995; Ozawa, 1997; Nielsen and Chuang, 2000):

(A1) $$\begin{array}{l} T\rho =\underset{j}{\sum }V_{j}^{*}\rho V_{j}, \end{array}$$

where (*V*_*j*_) are some operators acting in *H*. Hence, for a (CP) instrument, we have: for each *a*_*i*_ ∈ *O*, there exist operators (*V*_*a*_*i*_*j*_) such that

(A2) $$\begin{array}{l} E\left(a_{i}\right)\rho =\underset{j}{\sum }V_{a_{i}j}^{*}\rho V_{a_{i}j}. \end{array}$$

Thus,

(A3) $$\begin{array}{l} \rho_{a_{i}}=\frac{\underset{j}{\sum }V_{a_{i}j}^{*}\rho V_{a_{i}j}}{\underset{j}{\sum }V_{a_{i}j}^{*}\rho V_{a_{i}j}}, \end{array}$$

where the trace one condition (Equation 48) implies that

(A4) $$\begin{array}{l} \underset{i}{\sum }\underset{j}{\sum }V_{a_{i}j}^{*}V_{a_{i}j}=I. \end{array}$$

The corresponding POVMs *M*_*a*_*i*__ can be represented as

(A5) $$\begin{array}{l} M_{a_{i}}=\underset{j}{\sum }V_{a_{i}j}^{*}V_{a_{i}j}. \end{array}$$

This is a really elegant mathematical representation. However, it might be that this mathematical elegance, and not a real physical situation, has contributed to widespread use of CP in quantum information theory (cf. Shaji and Sudarshan, 2005).

Is the use of the extendability postulate justified in the operational approach to cognition?

Seemingly, not (although further analysis is required). Any concrete perception takes place at the conscious level, and it is based on interaction with the sensation of a visual image. The state of this sensation corresponds to the state of the system *S* in the above considerations. To be able to consider the state of another sensation, the analog of the state of the system $\tilde{S},$ the brain has to *activate* this sensation. Thus, we cannot simply consider all possible sensations as existing in some kind of the mental universe simultaneously. Hence, in general, sensations generated by different visual stimuli cannot be treated as existing simultaneously.

It is more natural to develop the theory of perception instruments as the theory of DL instruments and not CP instruments. In particular, although the Kraus representation can be used as a powerful analytic tool, we need not to overestimate its applicability for modeling of cognition.
